# Editorial: Transforming dementia caregiving through assistive technologies

**DOI:** 10.3389/fmed.2026.1864422

**Published:** 2026-05-14

**Authors:** Quan Zhang, Yijin Wu

**Affiliations:** 1 Ocean University of China, School of International Affairs and Public Administration, Qingdao, China; 2 Shandong University, Center for Quality of Life and Public Policy, Qingdao, China; 3 Qufu Normal University, Center for Geriatric Heathcare Services and Health Education, Rizhao, China

**Keywords:** caregiver support, dementia, early detection methods, integrated care models, technology-assisted interventions

## Introduction

As populations age globally, dementia represents an increasing healthcare challenge, particularly among older adults (1). Its growing prevalence underscores the need for effective caregiving approaches that support both individuals living with dementia and their families. Assistive technologies have been introduced as a means of complementing conventional care, providing additional options for monitoring, engagement, and support (2). Evidence suggests that such tools may contribute to improved quality of life, greater independence, and enhanced safety for people with dementia, while also helping to reduce caregiver burden (3, 4). The studies included in this Research Topic address a range of topics, including early detection methods, integrated care models, technology-assisted interventions, caregiver support, and the role of artificial intelligence in Alzheimer's disease research and care. Taken together, they offer a structured overview of current developments in technology-supported dementia care.

## Emerging technologies for early detection and diagnosis

Advances in sensing technologies and computational methods are facilitating earlier and more precise identification of dementia-related symptoms. Malioukis et al. reviewed wearable sensor systems for detecting agitation, showing that multimodal devices combined with individualized machine-learning approaches can identify agitation symptoms in real time. In parallel, developments in imaging analysis have improved diagnostic precision. Maity et al. employed deep learning models (DeepLabV3+, Deep Residual U-NET) to segment hippocampal and ventricular regions from structural and functional MRI data, integrating these outputs with hybrid classification methods. Their approach demonstrates high accuracy in the early detection of Alzheimer's disease. In addition, Fairburn et al. examined the potential role of quantum computing in medicine, noting its possible contributions to diagnostic accuracy and computational efficiency. Overall, these studies indicate that digital biomarkers, AI-assisted imaging, and emerging computational frameworks are increasingly being combined to support earlier diagnosis and ongoing monitoring in dementia care.

## Integrated and holistic care approaches

Multicomponent care models that combine established clinical practices with technological support are gaining attention in dementia care. Fang et al. evaluated an Alzheimer's care program combining person-centered assessment, cognitive and exercise therapies, structured communication, caregiver support, and digital health integration for cognitive training and monitoring. Their findings suggest that integrating assistive technologies within established care frameworks may improve patient outcomes and help alleviate caregiver burden. More broadly, such technology-supported, integrated approaches represent a developing direction in the management of Alzheimer's disease.

## Technology-enabled therapeutic interventions

Assistive technologies also serve as platforms for delivering non-pharmacological interventions. Yang et al. conducted a cluster randomized controlled trial across 16 community centers in Taiwan and found that a 16-week video-guided multimodal program was associated with a significant reduction in appetite and eating disturbances among persons with dementia, symptoms that are often challenging to manage in advanced stages of the condition. In a related effort, Alarcão et al. used a user-centered design approach to develop KeepsakeBox, a digital platform intended to support the implementation of reminiscence therapy. Together, these studies suggest that carefully designed, technology-supported interventions may contribute to improvements in both the daily experience and functional outcomes of individuals with dementia.

## Empowering and supporting dementia caregivers

Technology also has an important role in supporting and educating caregivers. The Age-it project (Trolese et al.) examined the needs of informal dementia caregivers in Italy and identified a substantial gap in accessible information and training from the point of diagnosis onward. The authors recommend the development of mobile-optimized, user-friendly e-learning platforms that provide personalized, accessible guidance tailored to caregiver needs. In addition to informational support, digital interventions may also address emotional challenges. Manevich explored the use of letter-writing to the deceased—an approach grounded in expressive writing within thanatology—among family caregivers experiencing pre-death grief. This work suggests that technology-mediated writing interventions may support adaptive coping processes and help maintain continuing bonds, highlighting an often underexplored aspect of caregiver wellbeing.

## Artificial intelligence in Alzheimer's disease

A broader review of the literature indicates that artificial intelligence is playing an expanding role in Alzheimer's disease research and care. Song et al. conducted a bibliometric analysis to map developments in this area, identifying key research themes such as disease stage classification, early diagnostic screening, risk assessment, and prediction of disease progression. These findings provide an overview of current research directions and may help inform future work on technology-supported approaches to dementia care.

## Conclusion

The contributions in this issue point to increasing integration of assistive technologies—including wearable devices, AI-based diagnostic tools, digital therapeutic interventions, and caregiver support systems—within dementia care. When combined with established, evidence-based practices, these approaches may support more proactive, individualized, and sustainable models of care for both individuals with dementia and their caregivers.
